# Supplementary material for: Learning and visual discrimination in newly hatched zebrafish
Source: iScience. 2022 Apr 22;25(5):104283. doi: 10.1016/j.isci.2022.104283 (PMC9092964; doi:10.1016/j.isci.2022.104283)
Supplement: Document S1. Figures S1–S3 [file mmc1.pdf]

iScience, Volume *25*

Supplemental information

Learning and visual discrimination

in newly hatched zebrafish

Maria Santacà, Marco Dadda, Luisa Dalla Valle, Camilla Fontana, Gabriela Gjinaj, and Angelo Bisazza

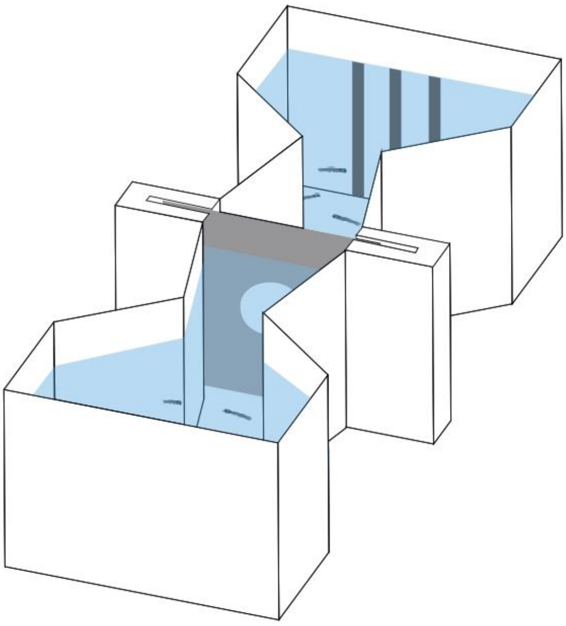

Figure S1. Apparatus used for the familiarization and group training phases, related to STAR Methods.

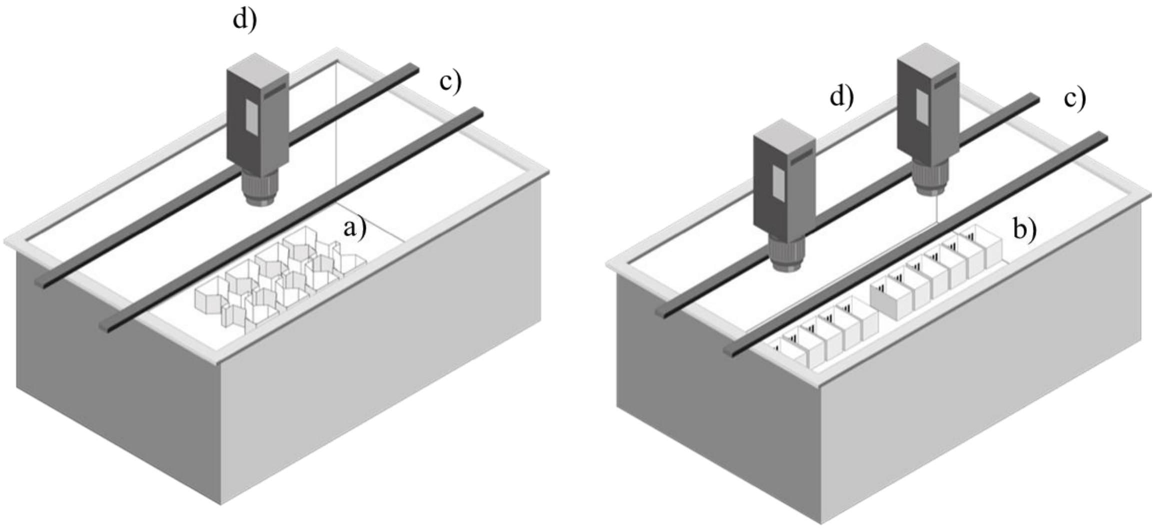

Figure S2. Setup for recording experiments, related to STAR Methods. Four identical group training apparatuses (a) and twelve individual apparatuses (b) were accommodated in opaque plastic box to reduce any external influence. The box was lit by two lamps (c) placed symmetrically along the major axis and behaviour was recorded by videocameras (d) placed above the box.

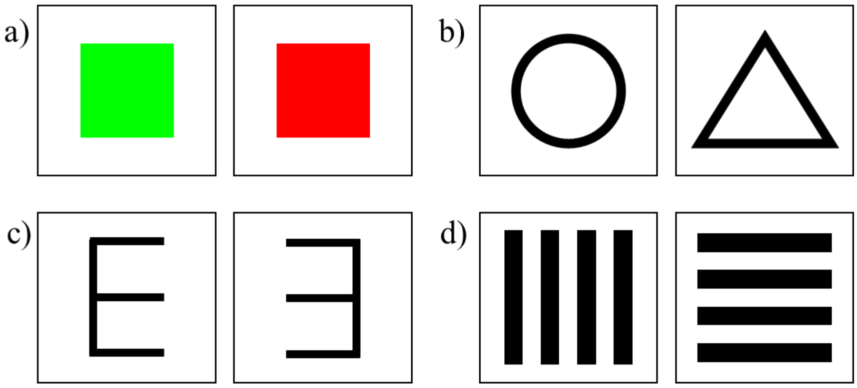

Figure S3, related to STAR Methods. Stimuli used in the colour discrimination (a), shape discrimination (b), mirror-image discrimination (c) and orientation discrimination (d).
